# Light at night acutely impairs glucose tolerance in a time-, intensity- and wavelength-dependent manner in rats

**DOI:** 10.1007/s00125-017-4262-y

**Published:** 2017-04-03

**Authors:** Anne-Loes Opperhuizen, Dirk J. Stenvers, Remi D. Jansen, Ewout Foppen, Eric Fliers, Andries Kalsbeek

**Affiliations:** 1 Hypothalamic Integration Mechanisms, Netherlands Institute for Neuroscience (NIN), Meibergdreef 47, 1105 BA Amsterdam, the Netherlands; 2 Laboratory of Endocrinology, Department of Clinical Chemistry, Academic Medical Centre (AMC) University of Amsterdam, Amsterdam, the Netherlands; 3 Department of Endocrinology and Metabolism, Academic Medical Center (AMC) University of Amsterdam, Amsterdam, the Netherlands

**Keywords:** Biological clock, Glucose intolerance, Green light, Insulin sensitivity, Light at night, Rat, Rodent

## Abstract

**Aims/hypothesis:**

Exposure to light at night (LAN) has increased dramatically in recent decades. Animal studies have shown that chronic dim LAN induced obesity and glucose intolerance. Furthermore, several studies in humans have demonstrated that chronic exposure to artificial LAN may have adverse health effects with an increased risk of metabolic disorders, including type 2 diabetes. It is well-known that acute exposure to LAN affects biological clock function, hormone secretion and the activity of the autonomic nervous system, but data on the effects of LAN on glucose homeostasis are lacking. This study aimed to investigate the acute effects of LAN on glucose metabolism.

**Methods:**

Male Wistar rats were subjected to i.v. glucose or insulin tolerance tests while exposed to 2 h of LAN in the early or late dark phase. In subsequent experiments, different light intensities and wavelengths were used.

**Results:**

LAN exposure early in the dark phase at ZT15 caused increased glucose responses during the first 20 min after glucose infusion (*p* < 0.001), whereas LAN exposure at the end of the dark phase, at ZT21, caused increased insulin responses during the first 10 min (*p* < 0.01), indicating that LAN immediately induces glucose intolerance in rats. Subsequent experiments demonstrated that the effect of LAN was both intensity- and wavelength-dependent. White light of 50 and 150 lx induced greater glucose responses than 5 and 20 lx, whereas all intensities other than 5 lx reduced locomotor activity. Green light induced glucose intolerance, but red and blue light did not, suggesting the involvement of a specific retina–brain pathway.

**Conclusions/interpretation:**

Together, these data show that exposure to LAN has acute adverse effects on glucose metabolism in a time-, intensity- and wavelength-dependent manner.

## Introduction

Electric lighting is widely used around the world, but possible harmful effects of the presence of light 24 h a day 7 days a week have become apparent only recently. In particular, the use of light at night (LAN) is considered potentially disruptive, as organisms are not physiologically prepared to deal with light signals during the night. Light is caught by the eyes and transformed into electrical signals by retinal receptors and conducted through the optic nerves to the brain areas important for vision, autonomic functions and circadian timing. Historically, sunlight would be mainly responsible for these functions, but artificial light with the appropriate wavelength and intensity may mimic the effects of sunlight.

Exposure to LAN has been correlated with an increased risk of developing obesity [1, 2], diabetes [3] and dyslipidaemia [2] in humans. In addition, rodent studies showed that chronic exposure to LAN reduces the amplitude of the sleep/wake rhythm [4–6], increases body weight and decreases glucose tolerance [4, 5]. Acute exposure to LAN has been shown to alter the profiles of the hormones melatonin and corticosterone [7, 8]. Moreover, LAN alters clock gene expression in the hypothalamic circadian clock [9], and affects gene expression in peripheral organs [7, 10]. Light is the most important cue for the circadian clock to synchronise its endogenously generated rhythmic activity with the environmental light/dark cycle. The clock, located in the suprachiasmatic nucleus (SCN), transmits timing information to downstream targets via hormones and the autonomic nervous system (ANS) to optimally prepare the organism for regular daily changes. For example, the SCN directly controls glucose homeostasis, including basal glucose levels and glucose tolerance [11, 12], at least partly through the ANS [13].

Visual and non-visual effects of light are mediated initially by retinal cells. Intense research into the non-visual effects of light revealed the existence of a novel retinal photoreceptor, the intrinsically photosensitive retinal ganglion cells (ipRGCs), which express the photopigment melanopsin [14, 15]. Knowledge of rods and cones is well established, and these cells are still considered to be the most important photoreceptors for vision, with rods being dominantly active in dim light and cones in bright light conditions. The ipRGCs provide the majority of retinal input to non-visual brain structures and dominantly innervate the SCN, providing information essential for circadian physiology [15]. However, the visual and non-visual pathways are not completely separate entities, as ipRGCs also receive indirect input from rods and cones [15–17].

The correlation between LAN and metabolic disorders together with the power of light to control the SCN and downstream targets led to the development of a hypothesis that LAN affects glucose homeostasis. The current study tested this hypothesis by subjecting Wistar rats to glucose and insulin tolerance tests during acute LAN at the beginning and end of the dark phase. As a secondary objective, we investigated glucose intolerance induced by acute LAN exposure in the early dark phase as a function of light intensity and wavelength.

## Materials and methods

### Animals and housing

Male Wistar rats (Charles River Breeding Laboratories, Sulzfeld, Germany) weighing 300–350 g were maintained under a controlled 12/12 light/dark cycle (lights on 07:00 hours, Zeitgeber Time 0 (ZT0); lights off 19:00 hours, ZT12), with ad libitum access to water and a regular chow diet (Teklad Global Diet, Harlan, Horst, the Netherlands), unless stated otherwise. Red dim light (max. 5 photopic lux [lx]) was present in the room during the dark phase, whereas mixed white fluorescent light (max. 150 lx) was present during the light phase. All experimental procedures were performed in accordance with the Council Directive 2010/63EU of the European Parliament and the Council of 22 September 2010 on protection of animals used for scientific purposes. All experimental procedures were approved by the Animal Ethics Committee of the Royal Dutch Academy of Arts and Sciences (KNAW, Amsterdam, the Netherlands) and in accordance with the guidelines on animal experimentation of the Netherlands Institute for Neuroscience.

### Experimental design

After adaptation to the animal facility, all rats, except the experimental groups for locomotor activity recordings, were anaesthetised with 80 mg/kg ketamine (Eurovet Animal Health, Bladel, the Netherlands), 8 mg/kg Rompun (xylaxine, Bayer Health Care, Mijdrecht, the Netherlands) and 0.1 mg/kg atropine (Pharmachemi, Haarlem, the Netherlands). Subsequently, an intra-atrial silicone catheter was implanted unilaterally in the jugular vein and fixed on the skull [12]. Animals were left to recover for at least 1 week before the start of experiments. To study whether LAN affects glucose or insulin tolerance, animals were exposed to 2 h of light during which an IVGTT or intravenous insulin tolerance test (IVITT) [12] was performed. Food was removed 2.5 h prior to the tests. In a randomly assigned crossover design, all animals included in the final data sets were tested twice, once under control (i.e. dim red light) and once under experimental (i.e. LAN) conditions, with a 1 week recovery period between. Animals with incomplete recovery of body weight or blockage of the catheter were excluded from the experiment.

### Experiment 1

Animals were randomly assigned to one of two groups to study the effects of LAN (white light ~125 ± 25 lx) on glucose tolerance: group 1A (*n* = 11) where under LAN conditions the light was turned on from ZT14–16 and the IVGTT started at ZT15, and under control conditions the IVGTT started at ZT15; and group 1B (*n* = 10) where under LAN conditions the light was turned on from ZT20–22 and the IVGTT started at ZT21, and under control conditions the IVGTT started at ZT21.

Two additional groups of animals were used to study insulin tolerance using an IVITT under the same light conditions as described for groups 1A and 1B. Group 1C (*n* = 13) was exposed to 2 h LAN from ZT14–16 and IVITT started at ZT15, and group 1D (*n* = 7) was exposed to 2 h LAN from ZT20–22 and IVITT started at ZT21.

### Experiment 2

Four groups were included to study the effect of different light intensities. The conditions were similar to experiment 1A: mixed white LAN from ZT14–16 and an IVGTT started at ZT15. Animals were randomly assigned to one of four groups: 5 lx (*n* = 10), 20 lx (*n* = 7), 50 lx (*n* = 9) or 150 lx (*n* = 9).

To study the effects of LAN on locomotor activity, four additional groups of animals were solitary housed in transparent cages to record locomotor activity with pressure plates [12]. Each measurement included 48 h continuous recording with 6 min intervals. In the first 24 h, locomotion was measured while animals were exposed to the normal (12/12) lighting schedule. In the second 24 h recording period, animals were exposed to LAN from ZT14–16. LAN exposure during this 2 h period was at 5 lx (*n* = 7), 20 lx (*n* = 5), 50 lx (*n* = 6) or 150 lx (*n* = 6).

### Experiment 3

Four groups were included to study the effects of wavelength. The conditions were similar to experiment 1A. Animals were randomly assigned to one of four groups and exposed to white light ranging from 400–700 nm (*n* = 9), blue light with a peak at 457 nm (*n* = 9), green light with a peak at 520 nm (*n* = 8) or red light with a peak at 633 nm (*n* = 10).

Four additional groups of animals were included as described in experiment 2 to study the effects of wavelength on locomotor activity. LAN exposure was the same as for the IVGTTs: white (*n* = 8), blue (*n* = 9), green (*n* = 9) or red (*n* = 9).

### Light exposure

White light exposure during experiments 1 and 2 was done with SMD3528 light-emitting diodes (LEDs; Watshome). Light exposure of different wavelengths in experiment 3 was done with SMD5050 LEDs (Watshome). Adhesive strips of LEDs were attached to in-house-built plastic walls surrounding the animal cages with horizontal light exposure at eye level of the animals. LEDs were controlled by software built in-house. The illuminance of the light for the different conditions in experiment 3 was adjusted such that photon flux and irradiance were similar between LAN conditions (see Table 1 for irradiance spectrum [18]).

**Table 1:** Spectral sensitivity of light conditions used in experiment 3

Retinal photopigment complement	White	Blue	Green	Red
S-cone (rN_sc_[λ])	0.41	1.46	0.17	0.08
Melanopsin (rN_z_[λ])	52.61	265.07	150.85	1.40
Rod (rN_r_[λ])	64.08	202.74	212.35	1.50
M-cone (rN_mc_[λ])	72.84	168.80	232.98	2.10
Irradiance (μW/cm^2^)	46.01	52.95	47.24	23.23
Photon flux (1 cm^−2^ s^−1^)	1.35 × 10^14^	1.28 × 10^14^	1.28 × 10^14^	0.74 × 10^14^

All data based on the rodent-toolbox provided by Lucas et al [18]

The upper rows represent weighted contribution of rodent retinal photopigments (S-cone, ipRGC [melanopsin], rod, M-cone) in α-opic rodent lux. The lower rows represent unweighted characteristics of the LEDs (irradiance, photon flux)

### Plasma measurements

Blood samples were cooled after collection, subsequently centrifuged (4°C, 1699 *g*, 15 min) and plasma was stored at −20°C until further analysis. Plasma glucose concentrations were determined immediately after blood sampling with a blood glucose monitoring system (FreeStyle Freedom-Lite, Abbott Diabetes Care, Alameda, CA, USA). Radioimmunoassays were used according to the manufacturer’s protocol to measure plasma insulin concentrations (Millipore, St Charles, MO, USA) and plasma corticosterone concentrations (MP Biomedicals, Santa Ana, CA, USA). Samples with incomplete duplicates due to technical reasons were excluded from the data.

### Statistical analysis

All data are expressed as means ± SEM. Paired *t* tests were used to detect group differences in baseline concentrations for glucose (experiments 1, 2 and 3), insulin (experiments 1 and 3) and corticosterone (experiments 1 and 3). Paired *t* tests were also used to detect group differences in locomotor activity in experiments 2 and 3. A repeated measures two-way ANOVA was used to test for effects of treatment (control or LAN), time or interaction (treatment × time) on the responses of glucose (experiments 1, 2 and 3), insulin (experiments 1 and 3) and corticosterone (experiments 1 and 3). If a treatment or interaction effect was found, post hoc Sidaks multiple comparisons tests were used to determine differences between light conditions at individual time points. The net AUC was determined from 0–60 min using the trapezoid rule. Delta values are calculated by subtracting the baseline (*t* = 0 min) from each time sample (*t* = 5, 10, 20, 30 and 60 min) raw data value. Paired *t* tests were used to detect group differences in AUC for glucose (experiments 1, 2 and 3), insulin (experiments 1 and 3) and corticosterone (experiments 1 and 3). All statistical analyses were performed with GraphPad Prism version 7.01 for Windows (GraphPad Software, La Jolla, CA, USA) using a significance level of *p* < 0.05.

## Results

### Experiment 1

**Fig. 1:**
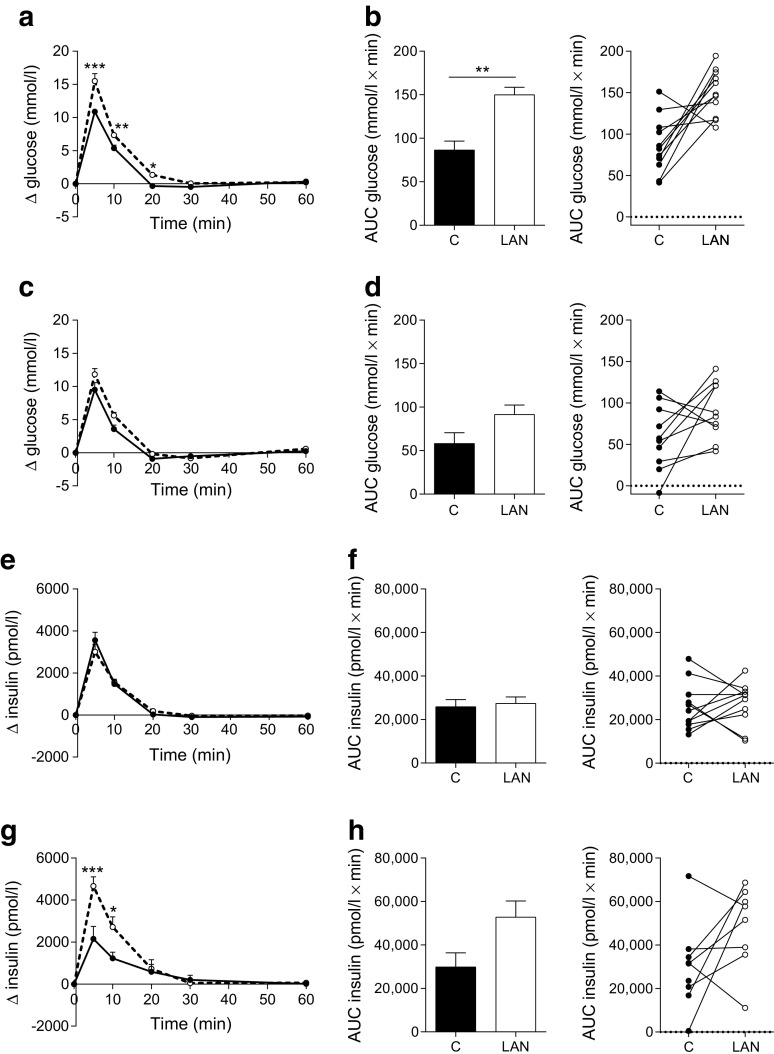
LAN decreases glucose tolerance at ZT15 and increases insulin responses at ZT21. LAN from ZT14–16 decreased glucose tolerance with higher glucose concentrations (**a**, **b**) and unchanged insulin responses (**e**, **f**). LAN from ZT20–22 did not affect plasma glucose concentrations (AUC *p* = 0.08) (**c**, **d**), but increased insulin concentrations (AUC *p* = 0.059) (**g**, **h**). AUC figures show the group mean on the left and individual animals on the right. Control (C), solid lines, black columns and symbols; LAN, dashed lines, white columns and symbols. *p < 0.05, **p < 0.01, ***p < 0.001

Effects of acute exposure to LAN on glucose homeostasis were tested by exposing rats to 2 h light at the beginning (ZT14–16) or end (ZT20–22) of the dark phase. In the early dark phase (ZT15), LAN induced increased plasma glucose levels after glucose infusion (Fig. 1a, b). No significant differences were observed for insulin (Fig. 1e, f). In the late dark phase (ZT21), LAN caused a trend towards increased plasma glucose (Fig. 1c, d). In contrast to ZT15, insulin plasma levels were increased by LAN at ZT21 after glucose infusion (Fig. 1g), resulting in a trend towards a higher AUC for insulin (Fig. 1h). Corticosterone responses were unaffected by LAN at ZT15 or ZT21 (Table 2).

**Table 2:** Statistical analyses of effects of light on glucose, insulin and corticosterone responses during glucose and insulin tolerance tests in experiment 1

Experiment	Variable	*p* for treatment	*p* for time	*p* for interaction	*p* for AUC
IVGTT					
ZT15	Glucose	0.002	<0.001	<0.001	0.003
	Insulin	0.769	<0.001	0.122	0.719
	Corticosterone	0.208	0.560	0.518	0.254
ZT21	Glucose	0.090	<0.001	0.063	0.080
	Insulin	0.015	<0.001	0.004	0.058
	Corticosterone	0.648	0.054	0.585	0.732
IVITT					
ZT15	Glucose	0.106	<0.001	0.298	0.118
	Corticosterone	0.532	<0.001	0.312	0.975
ZT21	Glucose	0.817	<0.001	0.250	0.617
	Corticosterone	0.061	0.114	0.240	0.069

Treatment (light condition), time (sample time) and interaction effects were determined using repeated measures two-way ANOVA

Glucose, insulin and corticosterone responses are reported for the IVGTT at ZT15 and ZT21. Glucose and corticosterone responses are reported for the IVITT at ZT15 and ZT21

The AUC column contains results of a paired *t* test on a net AUC curve

**Fig. 2:**
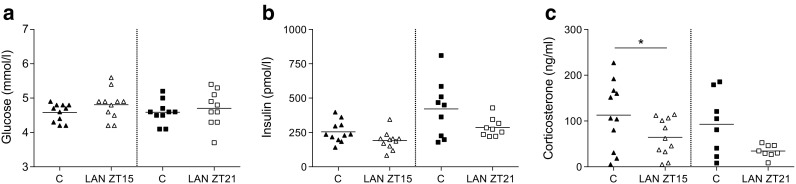
LAN decreases baseline corticosterone at ZT15 but does not affect baseline glucose and insulin concentrations. No significant differences were observed in baseline glucose (**a**) and insulin concentrations **(b**) between control (black symbols) and LAN (white symbols) conditions during experiment 1. LAN significantly decreased basal corticosterone concentrations at ZT15, but not at ZT21 (**c**). *p < 0.05

Tolerance tests were initiated at the start of the second hour of the light exposure; therefore, baseline samples (*t* = 0 min) were obtained after 1 h light exposure (both ZT15 and ZT21). Baseline glucose concentrations were unaffected by 1 h LAN at both time points (Fig. 2a). Baseline insulin concentrations tended to be lower both at ZT15 and ZT21 (Fig. 2b). Baseline levels of corticosterone were slightly reduced by LAN at ZT15 and showed a trend towards decreased levels at ZT21 (Fig. 2c).

**Fig. 3:**
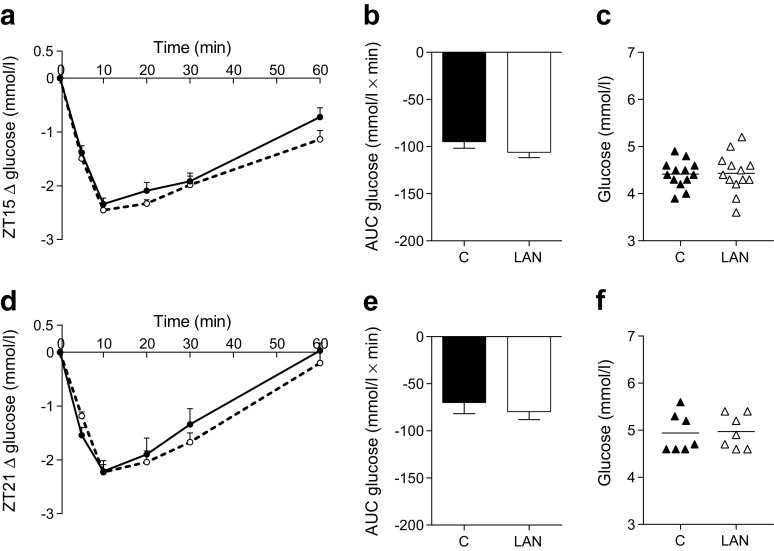
Blood glucose concentrations are unaffected by LAN during IVITT at ZT15 and ZT21. LAN did not alter the decrease in blood glucose after insulin infusion at ZT15 (**a**, **b**) or ZT21 (**d**, **e**). Baseline concentrations were not affected by LAN at ZT15 (**c**) or ZT21 (**f**) when compared with control conditions. Control (C), solid lines, black columns and symbols; LAN, dashed lines, white columns and symbols

To investigate whether LAN affects insulin sensitivity, animals were exposed to an IVITT. LAN did not induce changes in plasma glucose concentrations after insulin infusion at ZT15 (Fig. 3a, b) or ZT21 (Fig. 3d, e). Also corticosterone responses were not different between control and LAN conditions. Baseline concentrations of glucose (ZT15 Fig. 3c, ZT21 Fig. 3f) and corticosterone (ZT15: 41 ± 9 in control vs 70 ± 19 in LAN, *p* = 0.232; ZT21: 41 ± 16 vs 61 ± 27, *p* = 0.388) were unaffected by LAN at both time points. The statistical analyses of experiment 1 are reported in Table 2.

### Experiment 2

**Fig. 4:**
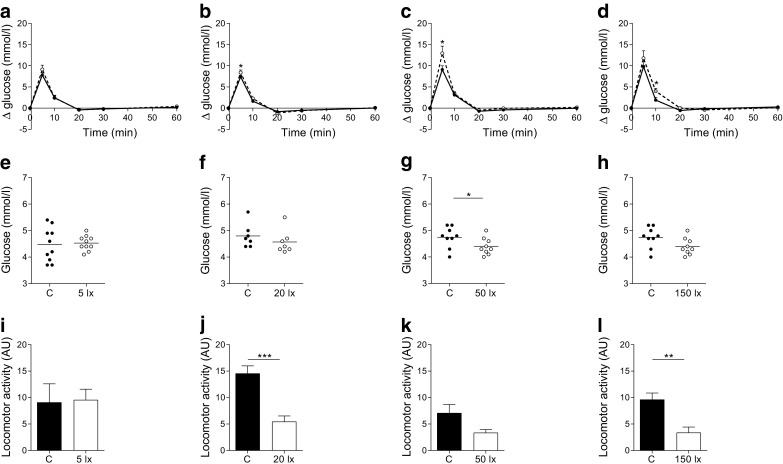
The effects of LAN on glucose tolerance and locomotor activity are intensity-dependent. LAN with intensity of 5 lx (**a**) did not induce changes in blood glucose concentrations, but 20 lx (**b**), 50 lx (**c**) and 150 lx (**d**) did. Baseline glucose levels were unaffected in 5 lx (**e**), 20 lx (**f**) or 150 lx (**h**) conditions, but slightly decreased by LAN of 50 lx (**g**). Total locomotor activity levels recorded from ZT14–16 were reduced during light conditions compared with control conditions for 20 lx (**j**), 50 lx (*p* = 0.05) (**k**) and 150 lx (**l**), but not 5 lx (**i**). Control (C), solid lines, black columns and symbols; LAN, dashed lines, white columns and symbols. *p < 0.05, **p < 0.01, ***p < 0.001

The effects of LAN on glucose tolerance were largest in the early dark phase (at ZT15) and therefore this time point was chosen to further investigate the characteristics of light**-**induced glucose intolerance. Four different light intensities were used to test whether the effects of light on glucose tolerance depend on intensity. Significantly higher peaks were found after glucose infusions in 50 and 150 lx conditions when compared with 5 and 20 lx (time *p* < 0.001; treatment [intensity] *p* = 0.013; interaction [intensity × time] *p* = 0.007). A significant treatment effect was only observed in the 50 and 150 lx conditions with higher glucose levels in LAN conditions when compared with control (Fig. 4a–d). AUC was significantly higher with 50 lx and tended to be at 150 lx. Baseline levels of glucose were unaffected by 5, 20 or 150 lx, and slightly reduced with 50 lx LAN (Fig. 4e–h). Locomotor recordings during LAN and control conditions showed decreased locomotion with 20, 50 and 150 lx. The decrease was significant for 20 and 150 lx (20 lx *p* = 0.001; 150 lx *p* = 0.007), with a similar trend for 50 lx (*p* = 0.052). Locomotor activity in animals exposed to 5 lx was unaffected (*p* = 0.915, Fig. 4i–l). The statistical analyses of experiment 2 are reported in Table 3.

**Table 3:** Statistical analyses of effects of light on glucose responses during glucose tolerance tests in experiment 2

Intensity	Variable	*p* for treatment	*p* for time	*p* for interaction	*p* for AUC
5 lx	Glucose	0.383	<0.001	0.078	0.524
20 lx	Glucose	0.362	<0.001	0.015	0.686
50 lx	Glucose	0.010	<0.001	<0.001	0.009
150 lx	Glucose	0.030	<0.001	0.073	0.106

Treatment (intensity), time (sample time) and interaction effects were determined using repeated measures two-way ANOVA

Glucose responses are reported for the IVGTT during four light intensities

The AUC column contains results of paired *t* test on the net AUC curve. The threshold for significance is *p* < 0.05

### Experiment 3

**Fig. 5:**
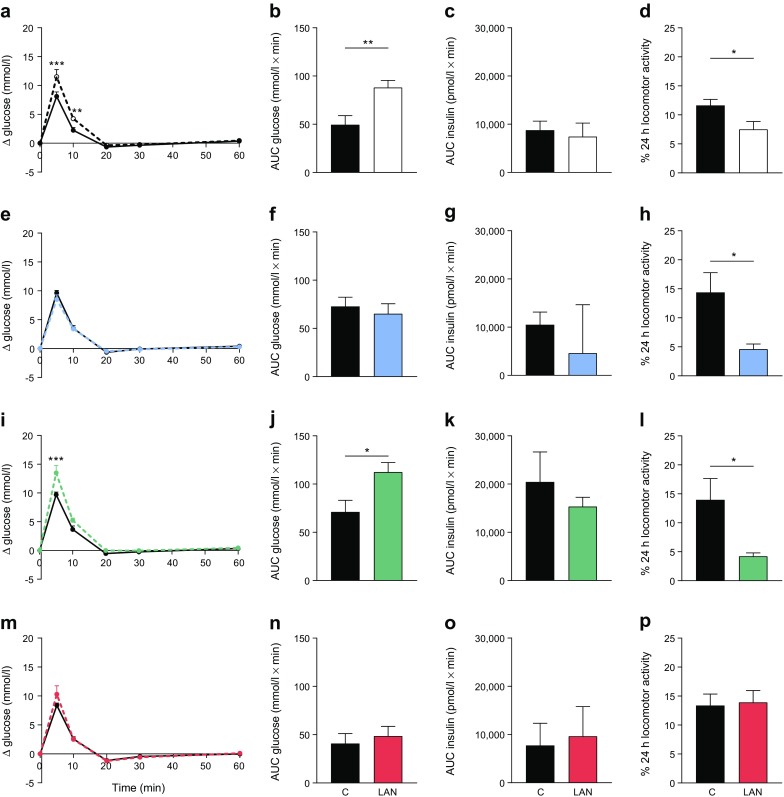
The effects of LAN on glucose tolerance and locomotor activity depend on wavelength. Two hours of white LAN increased glucose concentrations (**a**, **b**), did not induce changes in the insulin response (**c**) and decreased total locomotor activity (**d**). Blue light did not affect glucose concentrations (**e**, **f**) or the insulin response (**g**), but decreased total locomotor activity (**h**). Green light exposure increased glucose concentrations (**i**, **j**), did not affect the insulin response (**k**) and decreased total locomotor activity (**l**). Red light showed no effects on glucose concentration (**m**, **n**), the insulin response (**o**) or total locomotor activity (**p**). Control (C), solid lines, black columns and symbols; LAN, dashed lines, white/coloured columns and symbols. *p < 0.05, **p < 0.01, ***p < 0.001

**Fig. 6:**
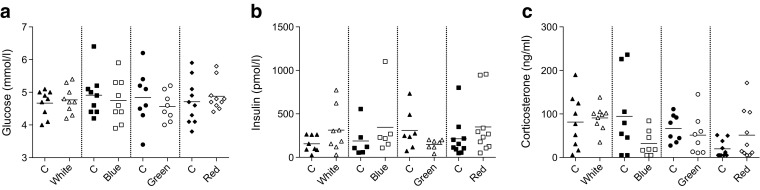
Baseline concentrations of glucose, insulin and corticosterone are unaffected by LAN at different wavelengths. No significant differences were observed in baseline glucose (**a**), insulin (**b**) and corticosterone (**c**) concentrations between control (black symbols) and LAN conditions (white symbols)

To study whether LAN-induced glucose intolerance was wavelength-dependent, four different wavelengths were used: short (blue), middle (green) and long (red), as well as broad-spectrum white light including all wavelengths (Table 1). As reported for experiments 1 and 2, white LAN induced glucose intolerance during a 2 h exposure starting at ZT14 (Fig. 5a, b). Increased glucose concentrations in the LAN condition were also observed in green light (Fig. 5i, j), but not in blue (Fig. 5e, f) or red light (Fig. 5m, n). Insulin responses were not affected by wavelength (Fig. 5c–o). Corticosterone responses were unaffected by blue, green or red light, but were significantly increased by white light exposure. Locomotor activity recordings demonstrated that white (*p* = 0.026), blue (*p* = 0.019) and green (*p* = 0.028), but not red (*p* = 0.812), light decreased locomotion compared with the control dark conditions (Fig. 5d–p). Baseline concentrations of glucose, insulin and corticosterone were unaffected by any of the light conditions (Fig. 6). The statistical analyses of experiment 3 are reported in Table 4.

**Table 4:** Statistical analyses of effects of light on glucose, insulin and corticosterone responses during glucose tolerance tests in experiment 3

Wavelength	Variable	*p* for treatment	*p* for time	*p* for interaction	*p* for AUC
White	Glucose	0.002	<0.001	0.018	0.009
	Insulin	0.616	<0.001	0.462	0.611
	Corticosterone	0.041	<0.001	0.114	0.008
Blue	Glucose	0.459	<0.001	0.154	0.602
	Insulin	0.609	<0.001	0.181	0.587
	Corticosterone	0.102	0.275	0.308	0.162
Green	Glucose	0.021	<0.001	0.001	0.027
	Insulin	0.428	<0.001	0.810	0.426
	Corticosterone	0.760	0.001	0.787	0.523
Red	Glucose	0.421	<0.001	0.172	0.603
	Insulin	0.375	<0.001	0.003	0.766
	Corticosterone	0.095	0.004	0.692	0.095

Treatment (wavelength), time (sample time) and interaction effects were determined using repeated measures two-way ANOVA

Glucose, insulin and corticosterone responses are reported for the glucose tolerance test in LAN conditions with four different wavelength spectra

The AUC column contains results of paired *t* test on the net AUC curve

## Discussion

We show that exposure to LAN causes acute glucose intolerance in rats and that this effect is dependent on the time of day, intensity and wavelength of the light exposure. The LAN-induced glucose intolerance at the start of the dark period was reflected by increased plasma glucose levels, whereas LAN-induced glucose intolerance at the end of the dark period was mainly reflected by increased plasma insulin. Surprisingly, green, but not blue, light best mimicked the effects of white light. These results suggest an important role for middle-wavelength cones in the LAN-induced effects on glucose intolerance.

Most non-visual light responses, such as the pupillary light response, melatonin inhibition and modulation of heart rate, are exerted via the ANS. Animal studies have shown that LAN acutely increases sympathetic activity and decreases parasympathetic activity of the autonomic nerves innervating peripheral organs, including the liver and pancreas [7, 19, 20]. Moreover, denervation of target organs, such as the liver [10] and adrenal gland [7], prevented LAN-induced changes in gene expression.

Here, we demonstrated that early LAN increased glucose levels without an accompanying increase in insulin response. In contrast, late LAN increased the insulin response with only small effects on the glucose response. These data suggest that LAN may affect glucose metabolism by several parallel mechanisms. LAN may have stimulated hepatic glucose production, which would explain the hyperglycaemia and is congruent with the reported upregulation of *Pepck* in the liver by LAN [10]. Stimulation of glucose production can lead to hyperglycaemia if the insulin response is inadequate. The discrepancy between the adequate insulin response at ZT21 and inadequate response at ZT15 indicates that the observed glucose intolerance at ZT15 is probably due to reduced beta cell sensitivity or an inhibition of insulin release. The inhibitory effect on insulin release seems to be reflected by the reduced baseline insulin concentration at ZT15 and ZT21. Such an inhibitory effect is in line with the previously reported light-induced reduction of glucose-stimulated insulin secretion [21] and the light-induced decreased parasympathetic and increased sympathetic input to the pancreas [20]. Moreover, in a recent study we found increased fasting and postprandial glucose levels as well as increased sympathetic activity in individuals with type 2 diabetes during early morning exposure to bright light [22].

Glucose intolerance could also have been caused by impaired insulin-independent and/or insulin-dependent glucose uptake by metabolic tissues. Little is known about the role of the ANS in glucose uptake, although it has been suggested that insulin-independent glucose uptake in adipose tissues and skeletal muscle depends on sympathetic innervation [23–26]. Whether light exposure stimulates sympathetic or parasympathetic innervation to muscles in a similar way as to the liver and pancreas [19, 20] has not been studied, and whether this would result in an increase or decrease in glucose uptake remains to be determined. However, the IVITT experiments showed that LAN did not reduce whole-body insulin sensitivity.

The discrepancy between insulin responses at the beginning and end of the dark period suggests that the SCN may modulate the light signal towards the pancreas in a time-dependent manner. Indeed, many other SCN-mediated effects of light have been shown to differ across the day [10, 27], and tracing studies have shown neural connections between the SCN and pancreas [28]. Hypothetically, light-induced SCN stimulation may also affect muscle function in glucose homeostasis, as it was reported that a daily rhythm exists in glucose uptake by muscle tissue in vitro [29], and disruption of the intrinsic muscle clock leads to the disturbance of glucose metabolism [30, 31].

One of the best-studied effects of LAN is the suppression of nocturnal melatonin release by the pineal gland. Experimental [32, 33] and genetic studies [34–36] have associated dysfunction of the melatonin system with glucose metabolism. Green and blue light exposure have both been shown to suppress melatonin secretion [37, 38]. However, the distinct glucose responses to LAN with blue and green light in this study make it unlikely that melatonin suppression is a major contributor to the observed glucose intolerance.

Melanopsin, the photosensitive pigment in ipRGCs, is most sensitive to short-wavelength light (~λ450 nm, the blue spectrum) and is responsible for phase-shifting [15], melatonin suppression [39] and arousal [40]. Our data, surprisingly, showed no effect of blue light on glucose metabolism. In contrast, green light clearly induced glucose intolerance. Therefore, our data do not support a direct role for the melanopsin pathway in the glucoregulatory effects of light. The human retina contains three types of cones: short (S-), middle (M-) and long (L-)cones, with M-cones being specifically sensitive to the green spectrum of light (λ520 nm). Data are limited on the physiological effects of green light and the role of M-cones, besides the suppression of melatonin secretion [37, 38] and involvement in circadian clock entrainment [41]. A recent study in mice [40] described distinct melanopsin-dependent effects of blue and green light on sleep, arousal and corticosterone release. The authors proposed that blue light stimulates M1-type ipRGCs projecting to the SCN and thereby acts on arousal and the ANS, whereas green light stimulates a different M-type of ipRGC that projects to the ventrolateral preoptic area and affects sleep. In line with this model, the effects of light on glucose metabolism in our study may be exerted via a non-M1 ipRGC subtype probably projecting preferentially to non-SCN target areas. Indeed, light-evoked effects on heart rate variability [42] and adrenal function [43] were also shown to be independent of ipRGCs innervating the SCN. Nocturnal rodents lack L-cones (λ_max_630 nm) and therefore do not show circadian, behavioural or metabolic effects in response to red light [37, 38, 44]. Our study is congruent with this as low illuminance (5 lx) and red light, main stimulators for L-cones, did not induce glucose intolerance. Furthermore, it is unlikely that S-cones play a significant role as none of our light sources stimulated these photoreceptors (Table 1). In addition to M-cones, rod photoreceptors also respond to green light and, therefore, a mediating role of rods in the effects of light on glucose tolerance cannot be excluded. However, the blue and green LEDs used in our study cause near-equal rod stimulation, whereas M-cone stimulation is higher with green light compared with blue light; therefore, we argue that M-cones are more important than rods in mediating the effects of LAN on glucose metabolism.

The inhibitory effects of LAN on behavioural activity in nocturnal animals are well-known and often reported as masking [45]. Inhibition of locomotor activity may result in reduced glucose uptake by muscle tissue; hypothetically explaining the observed glucose intolerance in this study. However, in view of the distinct responses of locomotion and glucose tolerance to blue light and low intensity white light, it is unlikely that the observed effects on glucose metabolism are mediated via this pathway.

Due to the widespread availability of artificial light, humans and animals around the world are increasingly exposed to LAN and knowledge on the metabolic effects of LAN is thus crucial. Disturbance of the circadian organised physiology by LAN is thought to lead to adverse metabolic health conditions in long-term studies [46]. Our study shows that LAN acutely affects the important homeostatic process of glucose tolerance. The effects on glucose and insulin suggest that light acutely affects the body at multiple levels downstream of the retina and hypothalamus. The nocturnality of rats and the diurnality of humans, as well as the differences in retinal sensitivity, should be taken into consideration when attempting to translate the findings in this study from animal to human. Results of three recent human studies (two performed during daytime and one at nighttime) are nicely in line with our current findings in demonstrating the potential of acute light exposure to affect glucose metabolism [22, 47, 48]. Therefore, chronic and acute light exposure now have been demonstrated to affect glucose metabolism in both rodent [4] and human studies [1–3, 22, 47, 48]. Chronic and acute LAN exposure are both representative conditions for human shift workers [46, 49], as well as for the global community exposed to increasing light pollution and the growing use of light-emitting products. Our study increases the knowledge of light effects on physiology, which may contribute to a better understanding of the correlation between LAN, shift work and metabolic disorders including diabetes.

In conclusion, we showed in rats that nocturnal light exposure acutely induced glucose intolerance, possibly via reduced beta cell sensitivity. This effect depended on the time of day, pointing towards an interaction with circadian physiology. The effect of light on glucose tolerance was also dependent on intensity and wavelength, suggesting a mediating role of non-M1 ipRGCs and M-cone photoreceptors. Our study warrants attention for the potentially hazardous actions of light on health in order to control the regulation of design and use of light-emitting products, along with advice for and protection of frequently exposed individuals.

## Abbreviations

ANS: Autonomic nervous system
ipRGC: Intrinsically photosensitive retinal ganglion cell
IVITT: Intravenous insulin tolerance test
LAN: Light at night
LEDs: Light-emitting diodes
SCN: Suprachiasmatic nucleus
ZT: Zeitgeber time
